# Genetic variation, structural analysis, and virulence implications of BimA and BimC in clinical isolates of *Burkholderia pseudomallei* in Thailand

**DOI:** 10.1038/s41598-024-74922-3

**Published:** 2024-10-23

**Authors:** Charlene Mae Salao Cagape, Rathanin Seng, Natnaree Saiprom, Sarunporn Tandhavanant, Claire Chewapreecha, Usa Boonyuen, T. Eoin West, Narisara Chantratita

**Affiliations:** 1 Department of Microbiology and Immunology, Faculty of Tropical Medicine, Mahidol University, Bangkok, Thailand; 2 Mahidol-Oxford Tropical Medicine Research Unit, Faculty of Tropical Medicine, Mahidol University, Bangkok, Thailand; 3 Parasites and Microbes, Wellcome Sanger Institute, Cambridge, UK; 4 Department of Clinical Tropical Medicine, Faculty of Tropical Medicine, Mahidol University, Bangkok, Thailand; 5 Department of Molecular Tropical Medicine and Genetics, Faculty of Tropical Medicine, Mahidol University, Bangkok, Thailand; 6 Division of Pulmonary, Critical Care & Sleep Medicine, Department of Medicine, University of Washington, Seattle, WA USA; 7 Department of Global Health, University of Washington, Seattle, WA USA

**Keywords:** *Burkholderia pseudomallei*, Melioidosis, BimA, BimC, Actin-based motility, Variation, Microbiology, Molecular biology

## Abstract

Melioidosis is a life-threatening tropical disease caused by an intracellular gram-negative bacterium *Burkholderia pseudomallei*. *B. pseudomallei* polymerizes the host cell actin through autotransporters, BimA, and BimC, to facilitate intracellular motility. Two variations of BimA in *B. pseudomallei* have been reported previously: BimA_Bp_ and BimA *B. mallei*-like (BimA_Bm_). However, little is known about genetic sequence variations within BimA and BimC, and their potential effect on the virulence of *B. pseudomallei*. This study analyzed 1,294 genomes from clinical isolates of patients admitted to nine hospitals in northeast Thailand between 2015 and 2018 and performed 3D structural analysis and plaque-forming efficiency assay. The genomic analysis identified 10 BimA_Bp_ and 5 major BimC types, in the dominant and non-dominant lineages of the *B. pseudomallei* population structure. Our protein prediction analysis of all BimA_Bp_ and major BimC variants revealed that their 3D structures were conserved compared to those of *B. pseudomallei* K96243. Sixteen representative strains of the most distant BimA_Bp_ types were tested for plaque formation and the development of polar actin tails in A549 epithelial cells. We found that all isolates retained these functions. These findings enhance our understanding of the prevalence of BimA_Bp_ and BimC variants and their implications for *B. pseudomallei* virulence.

**Supplementary Information:**

The online version contains supplementary material available at 10.1038/s41598-024-74922-3.

## Introduction

*Burkholderia pseudomallei* is the causative agent of melioidosis. It is a gram-negative intracellular soil-dwelling bacterium^1^ isolated from various soil and water sources in Thailand, Australia, and other tropical countries^2,3^. Routes of infection include direct aerosol inhalation, ingestion, and percutaneous inoculation^4^. Melioidosis has various clinical manifestations ranging from acute infections with pneumonia, sepsis, and disseminated internal abscess to localized and neurological infections^5,6^. The prevalence of melioidosis in Southeast Asia ranges from 0.02% to 74.4%, while the disease is estimated to affect 165,000 people worldwide of which 89,000 are fatalities^7,8^. Moreover, the fatality rate of melioidosis reaches 35%–40% in northeast Thailand^8,9^ and 10% in northern Australia^10^. The treatment regimen for melioidosis includes ceftazidime and meropenem for the initial parenteral phase, while trimethoprim/sulfamethoxazole (cotrimoxazole) is administered orally during the eradication phase^11^. *B. pseudomallei* is classified as a tier 1 select biological agent by the Centers for Disease Control and Prevention, posing a severe threat to both humans and animals^12,13^. Currently, licensed melioidosis vaccines and better treatment approaches are unavailable.

*B. pseudomallei* can invade and replicate inside phagocytic and nonphagocytic mammalian cells^14^. *B. pseudomallei* employs various virulence factors, such as Bsa (T3SS-3), to escape from vacuoles and survive in the cytoplasm^15^. When *B. pseudomallei* is inside the cytosol, it utilizes a motility factor, BimA^16^ and mimics the host cell actin nucleator, Ena/VASP, to polymerize actin, drives actin-based motility, and initiates host cell fusion^17^. BimA is a member of the trimeric autotransporter family and homologous to YadA of *Yersinia enterocolitica*^18^. BimA can induce actin-based membrane protrusions by polar nucleation^16,19,20^. When the protrusions connect with a neighboring cell, *B. pseudomallei* moves from one cell to another, spreads intracellularly, and fuses with neighboring cells, leading to form multinucleated giant cells (MNGCs) and plaques^19^. BimA is involved in the replication and intercellular spread of *B. pseudomallei* in many cell types, including epithelial^19^, macrophage-like^21^, and neuroblastoma^22^ cells. BimA is present in *B. pseudomallei* and closely related species, *B. mallei* and *B. thailandensis*^23^. *B. mallei* causes glanders, which primarily affects animals and can also infect humans. It is a clonal descendant of *B. pseudomallei*, having undergone genome reduction^24,25^. Although *B. thailandensis* is usually nonpathogenic to humans and animals, it has occasionally been isolated from humans and observed to be virulent in an insect model^26–28^. BimA of *B. mallei* and *B. thailandensis* can compensate for the actin-based motility function of the *bimA* knock-out mutant of *B. pseudomallei*^29^. There are two known types of BimA in *B. pseudomallei*: the typical BimA *B. pseudomallei* (BimA_Bp_) and the BimA *B.* mallei-like variation (BimA_Bm_). BimA_Bm_ is 54% identical to the BimA of *B. pseudomallei* K96243 (BimA_Bp_) and 95% identical to *B. mallei* ATCC 23344, respectively^23^. Studies have reported the BimA_Bm_ variants in Australian and South Asian isolates are associated with neurological melioidosis^30–32^. In an Australian study, 26% of 76 clinical isolates harbored BimA_Bm_, which was also linked to meropenem resistance and had a truncated *bimC* gene^32^. The BimA_Bm_ variant was observed in 4.5% and 18.5% of the isolates in India and Sri Lanka, respectively^30,31^; however, none were observed in 4 and 99 isolates of *B. pseudomallei* from Malaysia and Thailand, respectively^23,33^. In a murine melioidosis model, the BimA_Bm_ variant was more virulent when delivered intranasally and subcutaneously and persisted longer within the phagocytic cells compared to BimA_Bp_^34^.

BimC, located upstream of *bimA* in *B. pseudomallei* chromosome 2, is involved in actin-based motility, MNGC formation, and plaque formation^21^. BimC is a member of the bacterial autotransporter heptosyltransferase (BAHT) family^35^ and shares sequence homology with TibC from enterotoxigenic *Escherichia coli*^36^. BimC of *B. thailandensis* directly interacts with the transmembrane domain of BimA to confer polar targeting of BimA through the iron-finger motif formed by the four cysteine residues of BimC (C371, C374, C390, and C402)^37^. However, Srinon et al. previously described a polar expression of BimA in *B. pseudomallei* even after *bimC* deletion^21^.

Although variations in BimA have been studied across Australia and South Asia, particularly the BimA_Bm_ variant and its potential implications for virulence, it is necessary to explore novel sequence variations within BimA and BimC and their potential impact on *B. pseudomallei* virulence, especially in hyperendemic regions, such as northeast Thailand and its neighboring countries. In this study, we performed a genomic analysis to identify novel variations in BimA and BimC. Our genomic study analyzed 1,294 clinical isolates of *B. pseudomallei* collected from patients with melioidosis prospectively recruited into a cohort study conducted at nine hospitals in northeast Thailand. We then constructed three-dimensional (3D) structural models to predict whether these variations alter the structures of BimA and BimC proteins. Furthermore, we conducted assays to assess plaque-forming efficiency and performed immunostaining and confocal microscopy to investigate whether the identified BimA variants were associated with changes in the actin-based motility of *B. pseudomallei.*

## Results

### BimA and BimC are conserved in *B. pseudomallei* isolates in Thailand

In our 3-year prospective cohort study on melioidosis, known as the DORIM study, conducted in nine hospitals in northeast Thailand^38^, we collected 1,294 clinical isolates of *B. pseudomallei.* This study utilized whole genome sequencing data from the DORIM study^39^ to examine the genetic variations of *bimA* and *bimC*. Using the Basic Local Alignment Search Tool (BLAST), we observed that only 1,195 isolates contained the full length of *bimA* nucleotide sequences, while all 1,294 isolates had complete *bimC* nucleotide sequences. This discrepancy arose due to limitations in the short-read sequencing method, which resulted in *bimA* gene fragmentation (Supplementary Data 1).

**Figure 1:**
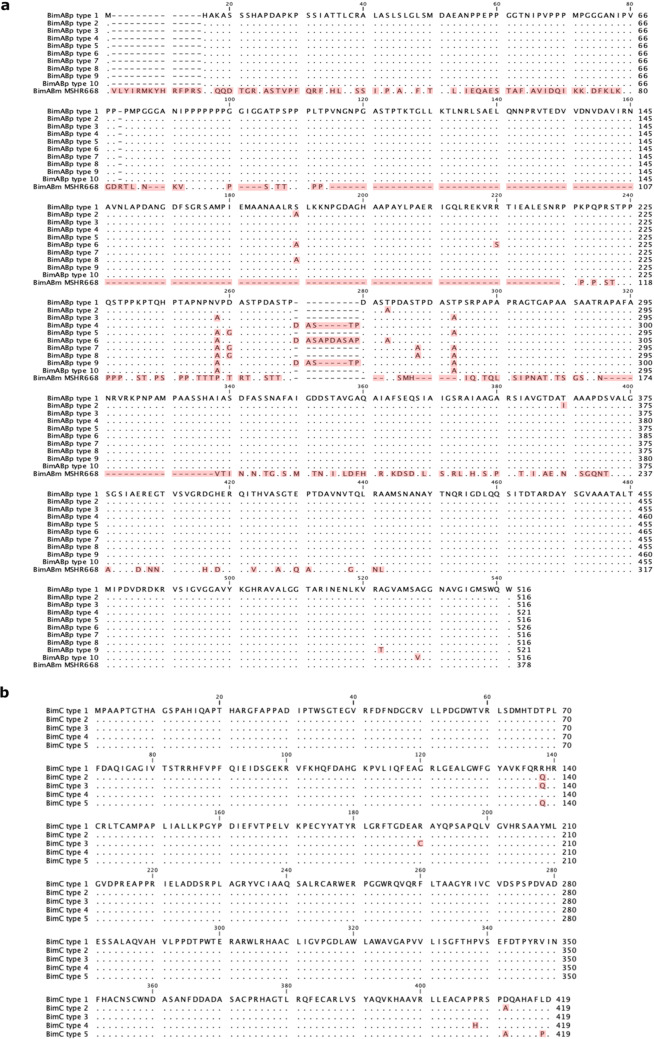
Alignment of the BimA_Bp_ and BimC types identified in clinical *B. pseudomallei* isolates. (**a**) Alignment of *B. pseudomallei* BimA_Bp_ types depicting the variations observed between types 1 and 10 compared to BimA_Bm_ MSHR668 ^23^ located at the bottom of the alignment. (**b**) Alignment of *B. pseudomallei* BimC types depicting the variations observed between types 1 and 5. *B. pseudomallei* K96243, classified as type 1 was used as the reference strain. Multiple sequence alignment was performed using ClustalW^40^.

**Figure 2:**
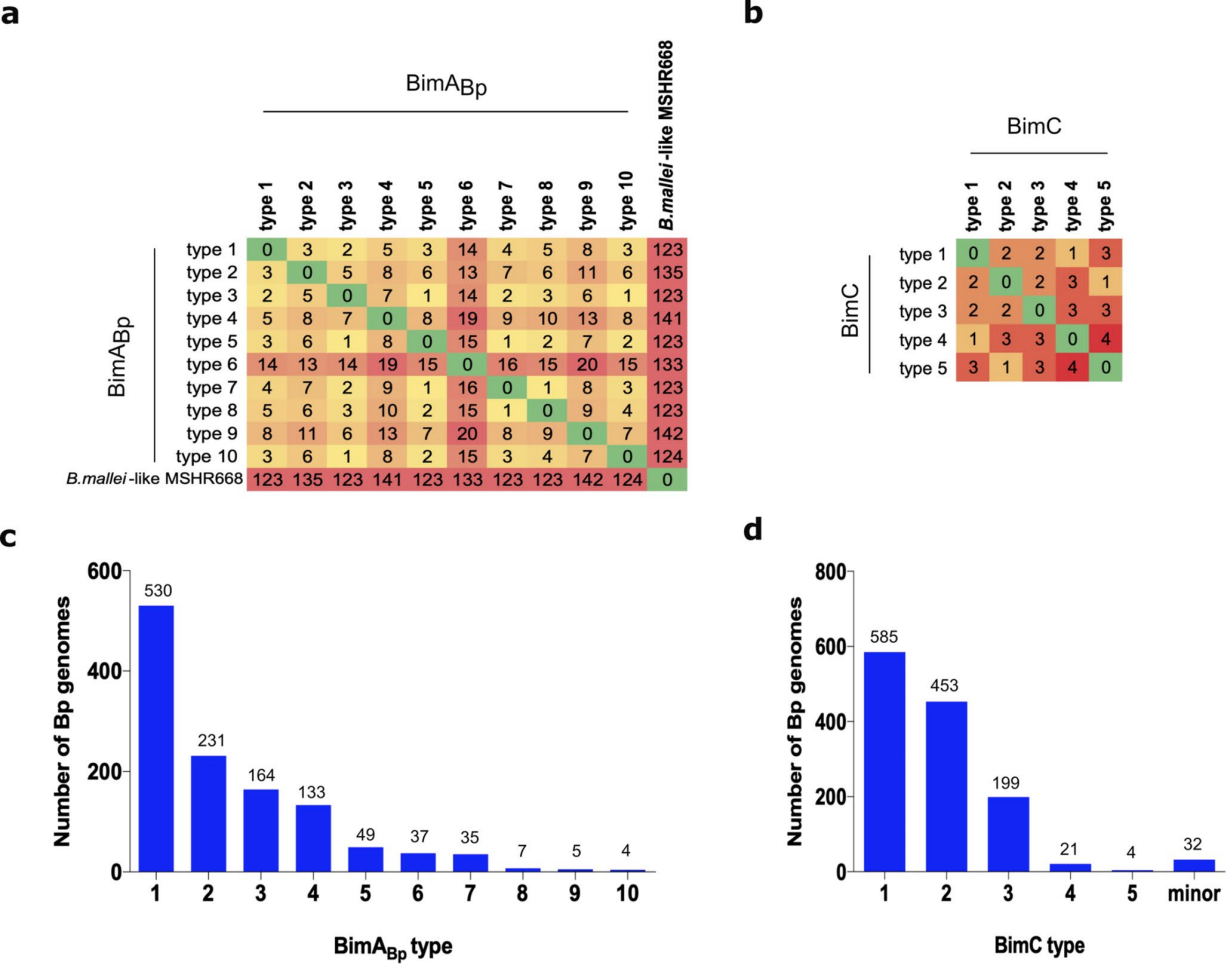
Number of *B. pseudomallei* genomes and Pairwise Single Amino acid Polymorphism (SAP) of BimA_Bp _and BimC types in clinical *B. pseudomallei* isolates. (**a**) and (**b**) Heat maps for pairwise SAP distances between BimA_Bp_ and BimC variants. The color and number correspond to the number of SAP distances between each variant type in reference to BimA_Bp_ and BimC types 1 (*B. pseudomallei* K96243, classified as type 1 was used as the reference strain). (**c**) and (**d**) Number of *B. pseudomallei* genomes harboring the BimA_Bp_ and BimC variant types.

Among 1,195 isolates of *B. pseudomallei*, none carried the *bimA*_*Bm*_ gene; however, all genomes carried *bimA*_*Bp*_, and variations were observed in this gene’s alleles. We then categorized BimA_Bp_ into different types. Genetic variants containing synonymous mutations after amino acid translation and have 100% amino acid sequence identity compared to *B. pseudomallei* K96243, were categorized as BimA_Bp_ type 1. Among all isolates with *bimA*_*Bp*_, nine BimA_Bp_ types diverged from BimA_Bp_ type 1 of *B. pseudomallei* K96243 (Figs. 1a and 2a and c; Table 1; Supplementary Data 3). Of the nine BimA_Bp_ types 2–10, six types (BimA_Bp_ types 2, 3, 5, 7, 8, and 10) possessed missense mutations compared to BimA_Bp_ type 1, while the remaining three types (BimA_Bp_ types 4, 6, and 9) had additional insertion sequences (Fig. 1a and Table 1). All the identified BimA_Bp_ types shared 97−99% sequence identity with BimA of the *B. pseudomallei* K96243.

**Table 1:** Variations detected in different types of BimA_Bp_ and BimC of *B. pseudomallei*.

Types of BimA and BimC	Number of genomes	Position	Mutation	% Sequence identity to K96243
BimA				
BimA_Bp_ type 1	530 (44.35%)	-	-	100
BimA_Bp_ type 2	231 (19.33%)	175	S175A	99.42
		258	T258A	99.42
		365	T365I	99.42
BimA_Bp_ type 3	164 (13.72%)	243	V243A	99.61
		268	T268A	99.61
BimA_Bp_ type 4	133 (11.13%)	244 − 248	244PDAST248	99.04
BimA_Bp_ type 5	49 (4.10%)	243	V243A	99.42
		245	D245G	99.42
BimA_Bp_ type 6	37 (3.10%)	175	S175A	97.34
		205	R205S	97.34
		243	V243A	97.34
		254 − 263	254PDAS*X*263	97.34
		263	T263A	97.34
		268	T268A	97.34
BimA_Bp_ type 7	35 (2.93%)	243	V243A	99.22
		245	D245G	99.22
		263	T263A	99.22
		268	T268A	99.22
BimA_Bp_ type 8	7 (0.59%)	175	S175A	99.03
		243	V243A	99.03
		245	D245G	99.03
		263	T263A	99.03
		268	T268A	99.03
BimA_Bp_ type 9	5 (0.42%)	243	V243A	98.66
		269 − 273	269PDAS*X*273	98.66
		273	T273A	98.66
		502	A502T	98.66
BimA_Bp_ type 10	4 (0.33%)	243	V243A	99.42
		268	T268A	99.42
		503	A503V	99.42
BimA_Bm_ MSHR668	0	2 − 15	Insertion	54
		18	K18Q	54
		19	A19Q	54
		20	S20D	54
		21	S21T	54
		22	S22G	54
		23	H23R	54
		25	P25A	54
		26	D26S	54
		27	A28T	54
		28	P28V	54
		29	K29P	54
		30	P30F	54
		31	S31Q	54
		32	S32R	54
		33	S33I	54
		35	T35H	54
		36	T36L	54
		39	R39S	54
		40	A40S	54
		41	L41I	54
		43	S43P	54
		45	S45A	54
		48	L48F	54
		50	M50T	54
		53	E53L	54
		55	N55I	54
		56	P56E	54
		57	P57Q	54
		58	E58A	54
		59	P59E	54
		60	P60S	54
		61	G61T	54
		62	G62A	54
		63	N63F	54
		65	I65A	54
		66	P66V	54
		67	V67I	54
		68	P68D	54
		69	P69Q	54
		70	P70I	54
		71	M71K	54
		72	P72K	54
		74	G74D	54
		75	G75F	54
		76	A76K	54
		77	N77L	54
		78	I78K	54
		81	P81G	54
		82	P82D	54
		83	Insertion	54
		84	P84T	54
		85	M85L	54
		87	G87N	54
		88 − 90	Deletion	54
		91	N91K	54
		92	I92V	54
		100	G100P	54
		101 − 104	Deletion	54
		105	A105S	54
		107	P107T	54
		108	S108T	54
		112	L112P	54
		113	T113P	54
		115 − 229	Deletion	54
		232	K232P	54
		234	Q234P	54
		236	R236S	54
		237	S237T	54
		241	Q241P	54
		242	S242P	54
		243	T243P	54
		246	K246S	54
		247	P247T	54
		249	Q249P	54
		250	H250S	54
		252	T252P	54
		253	A253P	54
		255	N255T	54
		256	P256T	54
		257	N257T	54
		258	V258P	54
		260	D260T	54
		261	A261R	54
		262	S262T	54
		265	D265S	54
		266	A266T	54
		267	S267T	54
		2821 − 282	Deletion	54
		285	D285S	54
		286	A286M	54
		287	S287H	54
		288 − 293	Deletion	54
		295	S295I	54
		296	R296Q	54
		298	A298T	54
		299	P299Q	54
		300	A300L	54
		302	R302S	54
		303	A303I	54
		304	G304P	54
		305	T305N	54
		306	G306A	54
		307	A307T	54
		309	A309T	54
		310	A310S	54
		311	S311G	54
		312	A312S	54
		315	R315N	54
		316 − 337	Deletion	54
		338	I338V	54
		339	A339T	54
		340	S340I	54
		341	D341N	54
		343	A343N	54
		345	S345T	54
		346	N346G	54
		348	F348S	54
		350	I350M	54
		352	D352T	54
		353	D353N	54
		355	T355I	54
		357	V357L	54
		358	G358D	54
		359	A359F	54
		360	Q360H	54
		362	I362R	54
		364	F364K	54
		365	S365D	54
		366	E366S	54
		367	Q367D	54
		369	I369L	54
		371	I371S	54
		373	S373R	54
		374	R374L	54
		376	I376H	54
		378	A378S	54
		380	A380P	54
		383	I383T	54
		385	V385I	54
		387	T386A	54
		388	D387E	54
		390	T390N	54
		392	A392S	54
		393	A393G	54
		394	P394Q	54
		395	D395N	54
		396	S396T	54
		400	S400A	54
		405	E405D	54
		407	E407N	54
		408	G408N	54
		416	D416H	54
		418	H418D	54
		423	T423V	54
		427	S427A	54
		430	E430Q	54
		431	P431A	54
		438	T438G	54
		441	R441N	54
		442	A442L	54
BimC				
BimC type 1	585 (45.21%)	-	-	100
BimC type 2	453 (35.00%)	138	R138Q	99.52
		412	D412A	99.52
BimC type 3	199 (15.38%)	138	R138Q	99.52
		190	R190C	99.52
BimC type 4	21 (1.62%)	408	P408H	99.76
BimC type 5	4 (0.31%)	138	R138Q	99.28
		412	D412A	99.28
		418	L418P	99.28

The analysis of *bimC*, when compared to *B. pseudomallei* K96243, revealed only missense mutations and lacked insertions or deletions. Based on the missense mutations, we categorized *bimC* into five major types (BimC types 1–5) and 25 minor types (BimC types 6–30) (Figs. 1b and 2b and d; Table 1; Supplementary Data 1 and Supplementary Data 4). BimC type 1 and types 2–5 shared 100% and more than 99% sequence identity with BimC of the *B. pseudomallei* K96243, respectively (Table 1). The 3D structures of all BimA_Bp_ and major BimC types were further characterized, and the representative isolates harboring the BimA_Bp_ types 2, 4, 6 and 9 (most distant from BimA_Bp_ type 1) and BimA_Bp_ type 10 (carrying a missense mutation in the transmembrane domain) were selected further for plaque-forming efficiency assay. These selections aimed to predict BimA-based functions in the virulence of *B. pseudomallei.*

### BimA_Bp _and BimC variants are specific to dominant *B. pseudomallei* lineages

**Figure 3:**
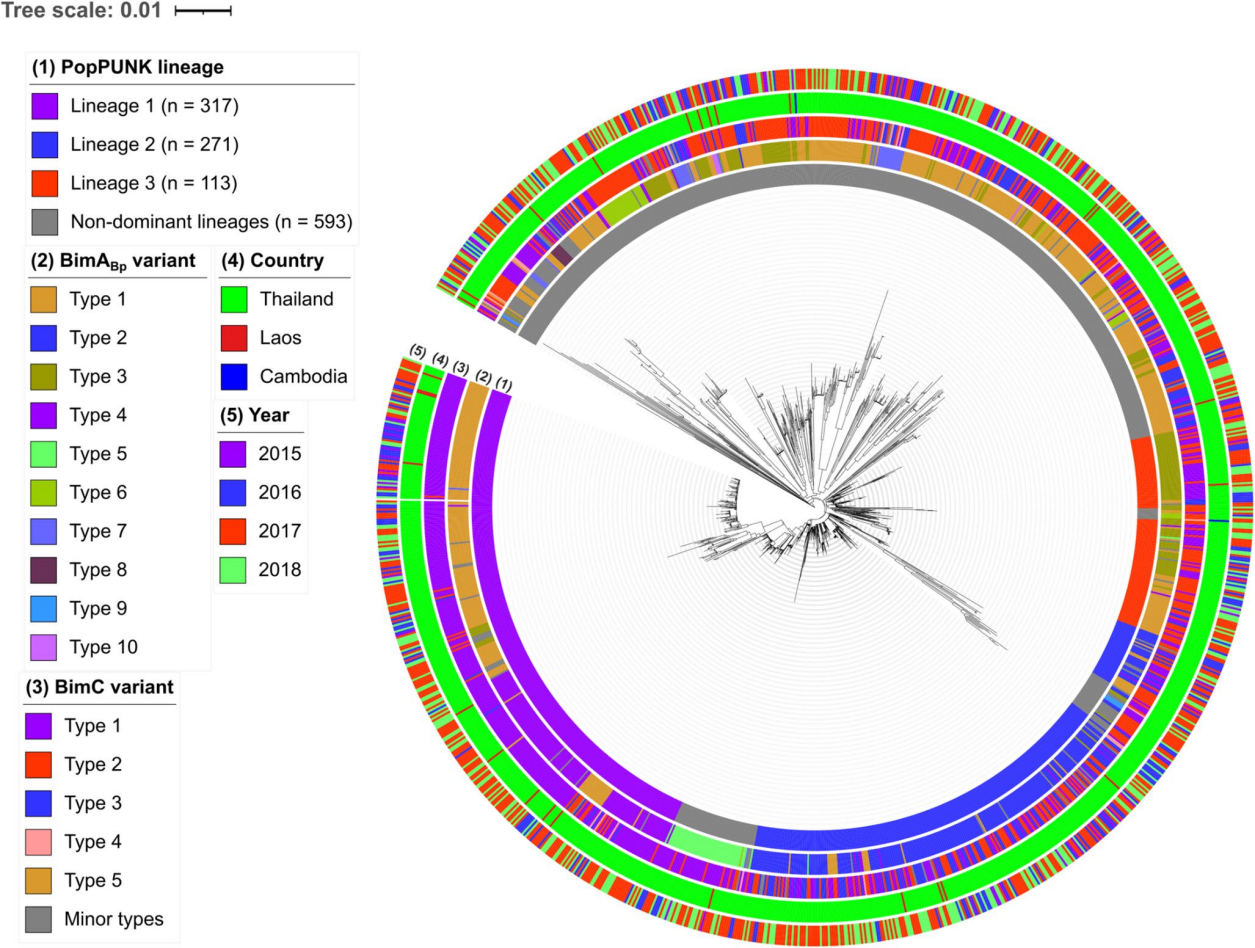
Maximum-likelihood phylogenomic tree of 1,294 *B. pseudomallei* clinical isolates rooted on MSHR5619. The innermost ring (1) represents the PopPUNK lineages. The second (2) and middle (3) rings represent the BimA_Bp_ and BimC variant types, respectively. The fourth (4) and outermost (5) rings represent the country sources based on patients’ home addresses and the year of sample collection, respectively. The tree scale indicates 0.01 nucleotide substitutions per site.

The population structure of *B. pseudomallei* in northeast Thailand was outlined from 1,265 genomes in our recent study^39^. To investigate BimA_Bp_ and BimC variants further, we incorporated 27 and 2 genomes from clinical isolates of Laos and Cambodian patients (Supplementary Data 1) into the existing 1,265 genomes. These patients were admitted to the study hospitals in Thailand^38^. Subsequently, we re-evaluated the population structure of the combined set of 1,294 genomes (Supplementary Data 1) using maximum-likelihood (ML) phylogeny and performed PopPUNK analysis. Consistent with our previous study^39^, our approach assigned 1,294 genomes into 101 lineages, which were classified into 3 dominant lineages (lineage 1, *n* = 317; lineage 2, *n* = 271; and lineage 3, *n* = 113), accounting for 54.2%, and 98 non-dominant lineages (lineages 4–101, *n* = 1–52), accounting for 45.8% (Fig. 3 and Supplementary Data 1 and Supplementary Table S1). Despite a limited number of isolates from Laos patients included in this study, *B. pseudomallei* from both Thailand and Laos displayed an inter-mixed pattern, as supported by the presentation of Laos (*n* = 14) in all three dominant lineages (Fig. 3). Moreover, our study revealed presentations of the dominant lineages, BimA_Bp_ and BimC types in each year of sample collection, which spanned from 2015 to 2018.

Our results in Fig. 3 and Supplementary Table S1 demonstrated that BimA_Bp_ types 1 and 4 were likely associated with lineage 1 (type 1, 55%, *P* = < 0.0001; type 4, 37%, *P* = < 0.0001), while BimA_Bp_ types 2 and 3 were likely specific to lineages 2 and 3, respectively (type 2, 83%; *P* = < 0.0001; type 3, 60%; *P* = < 0.0001). On the other hand, BimC type 1 was distributed across all lineages but was predominantly enriched in lineage 1 (90%; *P* = < 0.0001). The remaining BimC types 2–5 were present in lineages 2, 3, and in non-dominant lineages.

### BimA_Bp_ and BimC variants are dispersed across the endemic areas

**Figure 4:**
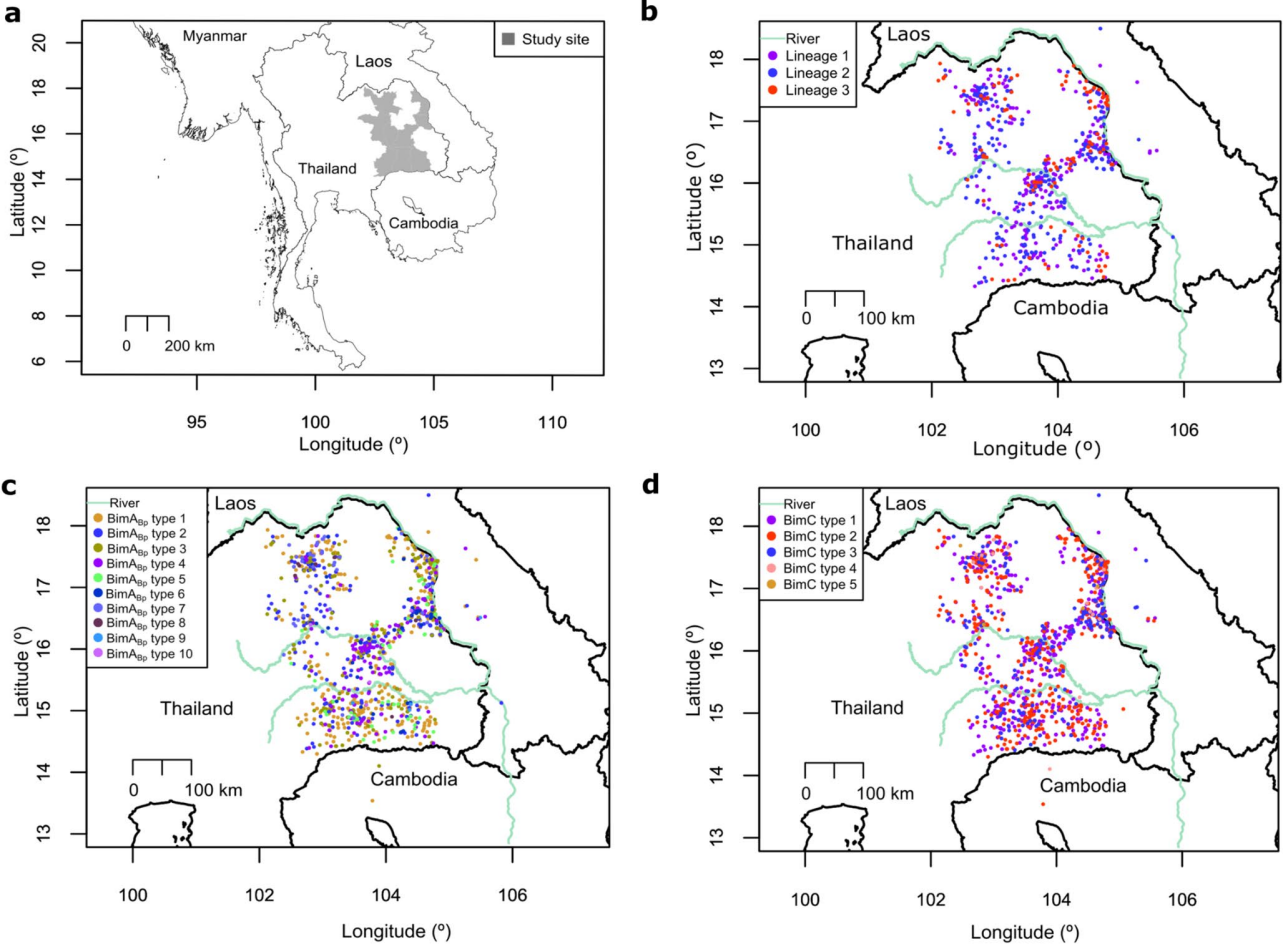
Geographical distribution of *B. pseudomallei* clinical genomes. (**a**) Geographical map of northeast Thailand with study sites highlighted in gray. (**b**) Geographical distribution of the three dominant PopPUNK lineages. (**c**) Geographical distribution of the ten BimA_Bp_ types. (**d**) Geographical distribution of the five BimC types. The spatial distribution of genomes was represented by the patients’ home addresses in Thailand, Laos and Cambodia.

We plotted the distribution of BimA_Bp_ and BimC variants in northeast Thailand, Laos, and Cambodia using the longitudinal and latitudinal coordinates obtained from the patients’ residences (Fig. 4). Dominant lineages 1–3 were noted in patients from northeast Thailand and Laos, consistent with the ubiquitous dispersal of dominant lineages (Fig. 4b) as described by Seng et al.^39^. Associated with the dispersal of lineages 1–3, the dominant BimA_Bp_ (BimA_Bp_ types 1–4) and BimC (BimC types 1–3) types were also ubiquitously present across the studied regions (Fig. 4c-d).

### 3D structural analysis of BimA_Bp_ types

**Figure 5:**
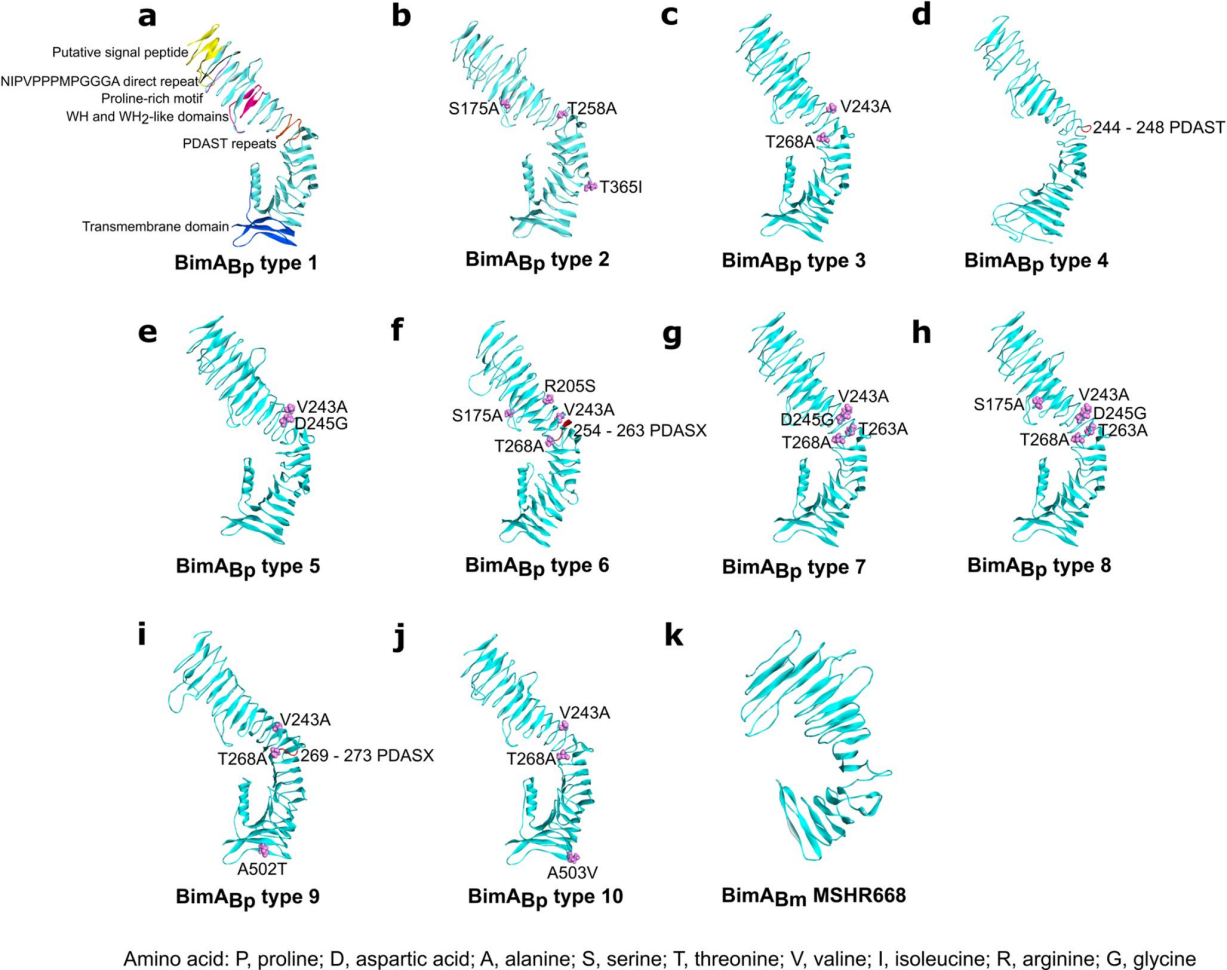
3D structural models of ten BimA_Bp_types and BimA_Bm_of Australian strain MSHR668 of *Burkholderia pseudomallei*. (**a**) 3D structural model of BimA_Bp_ type 1 (*B. pseudomallei* K96243, classified as type 1 was used as the reference strain) as described by Stevens et al.^16^. The yellow-colored ribbon represents the predicted signal peptide (residues 1–53); green, NIPVPPPMPGGGA direct repeat (residues 63–75); violet, proline-rich motif (residues 78–84); pink, WH and WH_2_-like domains (residues 155–158); orange, PDASX repeats (residues 244–268); blue, transmembrane domain (residues 458–516). (**b – j**) 3D structural models of BimA_Bp_ types 2, 3, 4, 5, 6, 7, 8, 9 and 10. Mutation positions are represented in pink Corey-Pauling-Koltun (CPK) models and labeled accordingly. The red-colored ribbons represent the insertion. (**k**) 3D structural model of BimA_Bm_ MSHR668 ^23^. All BimA_Bp_ and BimA_Bm_ models were built using I-TASSER. The C-score of the models were: -0.96 for BimA_Bp_ type 1; -1.16 for type 2; -0.71 for type 3; -0.74 for type 4; -0.99 for type 5; -0.73 for type 6; -1.02 for type 7; -1.12 for type 8; -1.13 for type 9; -0.71 for type 10; and  -0.93 for BimA_Bm_ MSHR668. Figures were generated by Discovery Studio Visualizer version 21.1.

We performed 3D structure modeling to predict the potential functional change of BimA_Bp_ variants (Fig. 5). The amino acid sequences of BimA *B. pseudomallei* K96243 were retrieved from GenBank (Reference: CAH38965.1, locus tag: BPSS1492) and subjected to a BLAST search against Protein Data Bank (http://www.rcsb.org) to identify an appropriate template for homology modeling. However, despite efforts to enhance the search using the Phyre 2 protein threading method^41^, a reliable template could not be identified due to low sequence identity and coverage. To overcome this limitation, we utilized the I-TASSER *de novo* protein modeling method^42^ to generate our own model, resulting in a model with a confidence score of -0.96. To ensure the model’s quality, we performed YASARA energy minimization^43^ and validated it using the SAVES PROCHECK server (https://saves.mbi.ucla.edu/). The 3D structure of the model was then visualized using Discovery Studio Visualizer software (Biovia v.21.1).

BimA *B. pseudomallei* is a type Vc trimeric autotransporter whose structure contains a C-terminal autotransporter domain anchored to the membrane by forming a pore and an N-terminal passenger domain, which transports protein and is exposed to the bacterial surface^44^. In reference to BimA_Bp_ type 1 of *B. pseudomallei* K96243 (Fig. 5a), the 3D structures of BimA_Bp_ types 2–10 did not differ despite the presence of single amino acid polymorphisms (SAPs) and insertions (Fig. 5b–j). The mutations found in each BimA_Bp_ type were listed in Table 1. Interestingly, BimA_Bp_ types 9 and 10 have amino acid amino acid changes in the transmembrane domain (458–516), which was previously described as a site of BimC interaction in *B. thailandensis*, crucial for the polar localization of BimA^37^.

The 3D structure of BimA_Bm_ has not been reported. Therefore, we constructed the 3D structural model of a BimA_Bm_ of an Australian strain MSHR668 (GenBank Ref.: NZ_CP009545) (Fig. 5k) using I-TASSER which resulted to model 1 with confidence score of -0.93 ^42^. Although the BimA_Bm_ variant shares only 54% of its sequence with BimA_Bp_ K96243 ^23^, its structure remains typical of an autotransporter in which the β-barrel C-terminal autotransporter domain serves as an anchor to the bacterial surface to form a pore for protein transport^18,45^.

### 3D structural analysis of major BimC types

**Figure 6:**
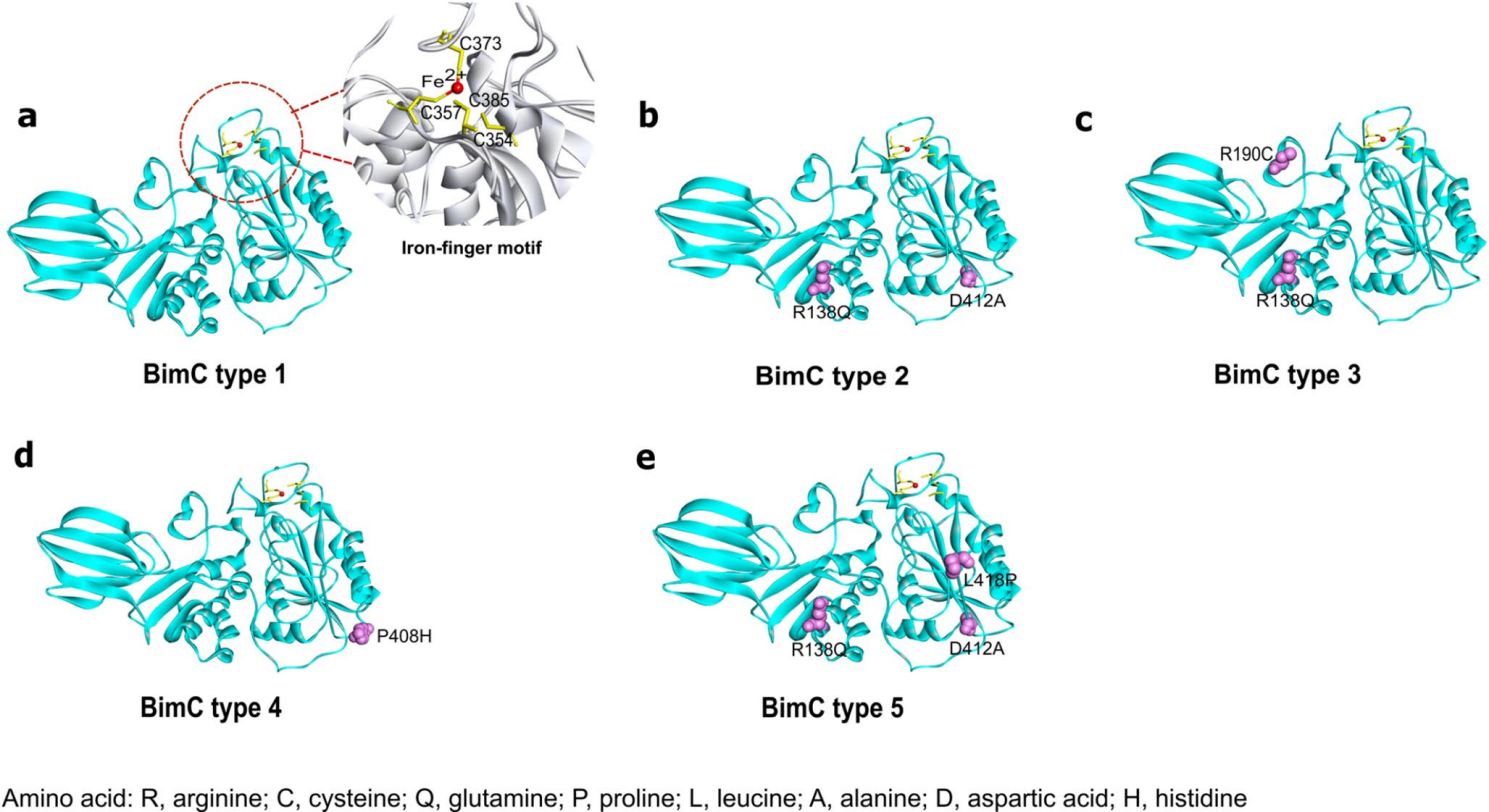
3D structural models of five major BimC types of *B. pseudomallei*. (**a**) The 3D structural model of BimC type 1 (*B. pseudomallei* K96243, classified as type 1 was used as the reference strain) was built based on a template TibC of *E. coli* H10407 (4RB4) using SWISS-MODEL, with 43.85% sequence identity and 93% coverage and GMQE score of 0.77. The iron-finger motif (C354, C357, C373 and C385) is shown as an inset. (**b – e)** 3D structural models of BimC types 2, 3, 4 and 5. Mutation positions are represented in pink Corey-Pauling-Koltun (CPK) models and labeled accordingly. Figures were generated by Discovery Studio Visualizer version 21.1.

The BimC amino acid sequence was retrieved from GenBank (Ref: WP_011205625.1, locus tag: BPSS1491) and categorized by Lu et al.^35^ into an autotransporter heptosyltransferase with a calculated molecular weight of 45,928 Dalton. The template for BimC homology modeling was based on SWISS-MODEL with GMQE (Global Model Quality Estimate) score of 0.77, using TibC, a dodecameric iron-containing heptosyltransferase from enterotoxigenic *E. coli* H10407 (4RB4), which has 43.85% sequence identity and 93% coverage of the BimC of *B. pseudomallei* K96243 ^46^. Visualization using Discovery Studio Visualizer software showed that our BimC variants possessed several missense mutations located in the alpha helices of the protein’s secondary structure and lacked insertion or deletion sequences (Fig. 6a and e). The mutations found in major BimC types were listed in Table 1.

Moreover, mutations were not found in the four cysteine residues (C354, C357, C373, and C385) in the sequences of BimC types 1–5. These cysteine residues bind to the iron ion (Fe^2+^) to form the unique iron-finger motif (Fig. 6a, inset)^35^. The iron-finger motif is essential for the polar targeting of BimC and polymerization of BimA in *B. thailandensis*^37^. These steps are necessary for actin-tail formation to enable bacterial movement within and between the host cells^47^. Although the variations in BimC were minimal, further investigation is needed to validate their involvement in actin-tail formation.

### *B. pseudomallei *isolates with different BimA_Bp _types can induce plaque formations

**Figure 7:**
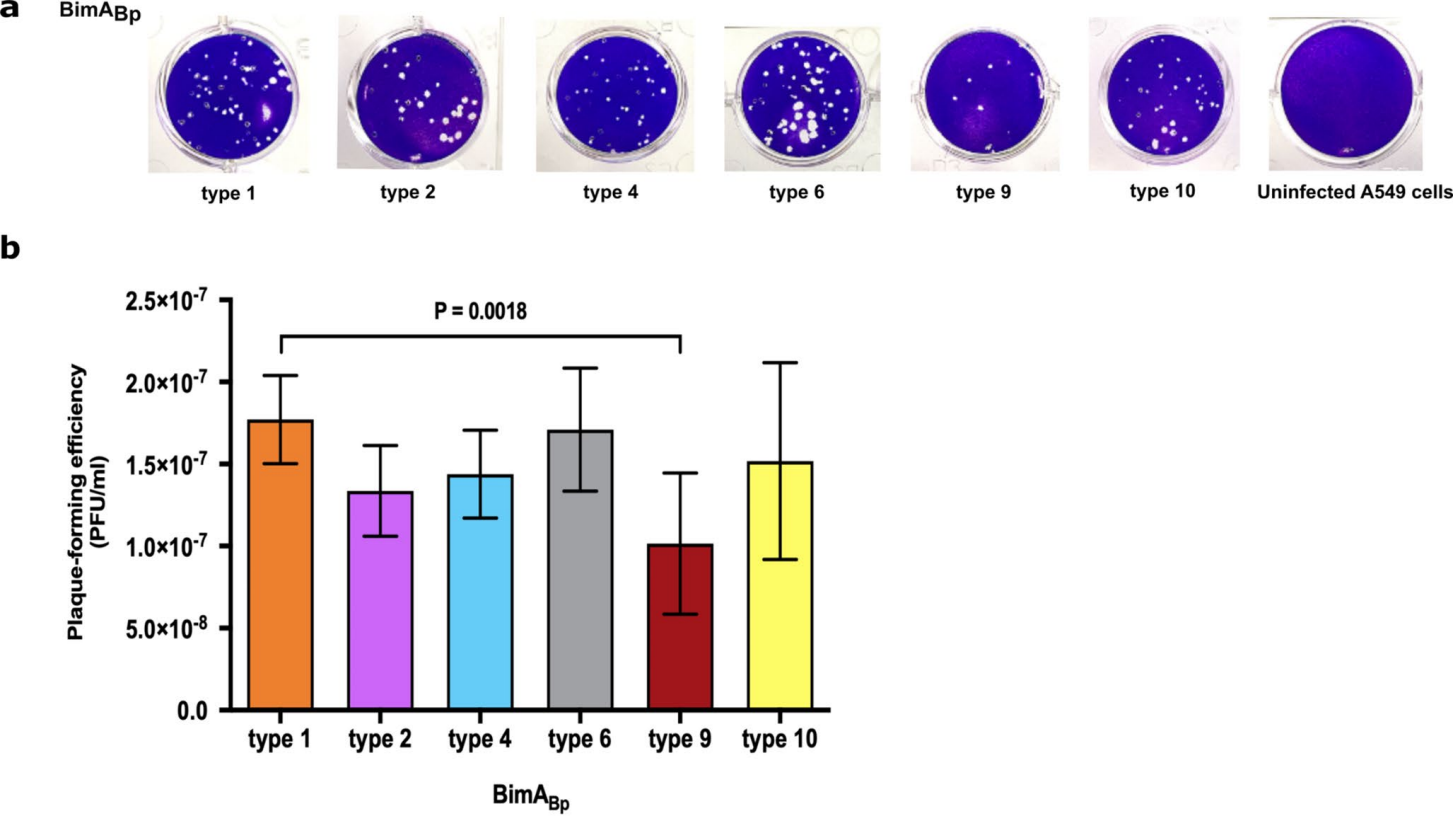
Plaque-forming efficiencies of representative strains harboring different BimA_Bp_types in A549 cells. (**a**) Photographic representation of plaques. (**b**) Plaque-forming efficiencies of *B. pseudomallei* isolates harboring the different BimA_Bp_ types in A549 cells. The cells were infected with *B. pseudomallei* strains representative of BimA_Bp_ type 1 (*B. pseudomallei* K96243, classified as type 1 was used as the reference strain), type 2 (DR10025A, DR20021A and DR40130A), type 4 (DR40111A, DR80025A and DR90085A), type 6 (DR10008A, DR40025A and DR50053A), type 9 (DR50173A, DR70003A and DR90006A), and type 10 (DR20062A, DR50003A and DR60054A) at MOI of 0.1:1. Plaques were stained with 2% (w/v) crystal violet at 24 h post-infection. Plaque-forming efficiency (PFU/ml) was counted as the number of plaques (plaque-forming units: PFU) divided by the CFU (colony-forming units) of bacteria added per well (CFU/ml). Error bars represent means ± standard deviation of data from three independent experiments (one-way ANOVA; *P* < 0.05).

Stevens et al. previously reported that *B. pseudomallei* uses BimA to facilitate its intracellular movement and host cell membrane protrusion by initiating host actin polymerization, thereby enabling bacterial spread from one cell to another^16^. A way to assess the effect of cell-to-cell spread is by observing the plaque-forming efficiency in infected cells^19^. In this study, we examined the plaque-forming efficiencies of representative isolates of BimA_Bp_ types 2, 4, 6, 9 (most distant from BimA_Bp_ type 1) and 10 (carrying a missense mutation in the transmembrane domain) compared to *B. pseudomallei* K96243 (BimA_Bp_ type 1) (Fig. 7). We observed plaques in A549 infected with the representative isolates although the plaque-forming efficiency (PFU/ml) of BimA_Bp_ type 2 (mean ± standard deviation (SD) = 1.34 × 10^−7^ ± 2.77 × 10^−8^ PFU/ml; *P* = 0.2053), BimA_Bp_ type 4 (mean ± standard deviation (SD) = 1.44 × 10^−7^ ± 2.68 × 10^−8^ PFU/ml; *P* = 0.4973), BimA_Bp_ type 6 (mean ± standard deviation (SD) 1.71 × 10^−7^ ± 3.75 10^−8^ PFU/ml; *P* = 0.9995) and BimA_Bp_ type 10 (mean ± standard deviation (SD) = 1.52 × 10^−7^ ± 6.00 × 10^−8^ PFU/ml; *P* = 0.7616) were not significantly different from that of BimA_Bp_ type 1 (K96243). Interestingly, BimA_Bp_ type 9 showed lower plaque-forming efficiency and was statistically different compared to BimA_Bp_ type 1 (K96243) (mean ± SD = 1.02 × 10^−7^ ± 4.30 × 10^−8^ PFU/ml versus 1.77 × 10^−7^ ± 2.69 × 10^−8^ PFU/ml; *P* = 0.0018) and other types. The lower plaque formation in BimA_Bp_ type 9 could be contributed by an amino acid change from threonine to alanine in the PDASX region, a predicted CK2 phosphorylation site that plays a role in actin polymerization and assembly^16,48^.

Furthermore, we examined if the sixteen strains used in plaque assay possessed variations in proteins (BPSL0097, BPSS0015, BPSS1494 (VirG), BPSS1495 (VirA), BPSS1498 (Hcp-1), BPSS1818 and BPSS1860) involved in plaque formation and actin-based functions, as described in previous studies^18,49–51^ (Supplementary Data 2). We found that variations in BPSL0097, BPSS0015, BPSS1494 (VirG), BPSS1495 (VirA), BPSS1498 (Hcp-1), BPSS1818 and BPSS1860 also exist in these genomes.

### *B. pseudomallei* isolates with different BimA_Bp _types are all capable of inducing actin tails

**Figure 8:**
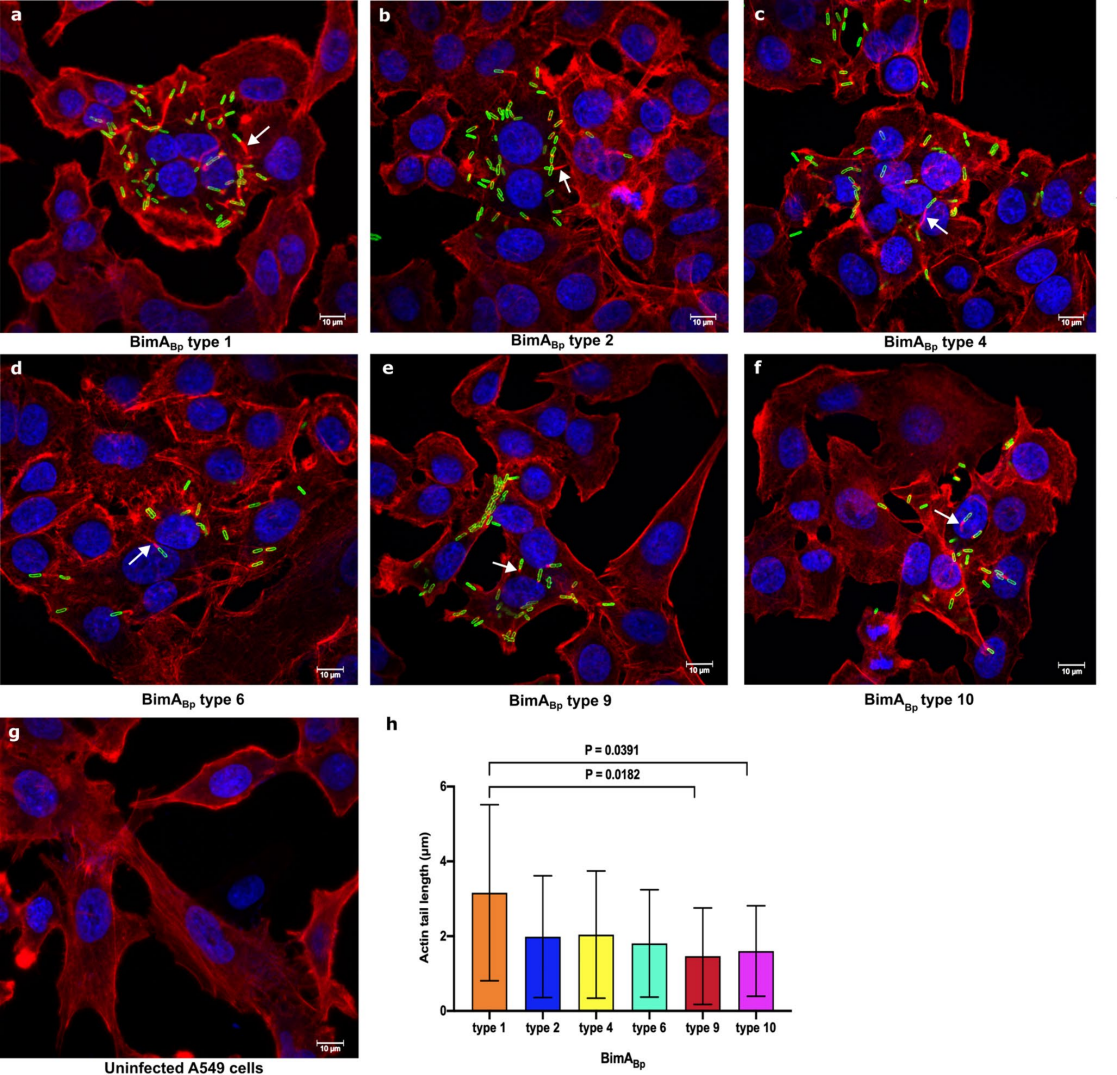
Confocal microscopy images of A549 cells infected with *B. pseudomallei* isolates with different BimA_Bp_types. The representative strains of *B. pseudomallei *(**a**) BimA_Bp_ type 1 (*B. pseudomallei* K96243, classified as type 1 was used as the reference strain), (**b**) BimA_Bp_ type 2 (DR40130A), (**c**) BimA_Bp_ type 4 (DR40111A), (**d**) BimA_Bp_ type 6 (DR10008A), (**e**) BimA_Bp_ type 9 (DR50173A), and (**f**) BimA_Bp_ type 10 (DR50003A) were used to infect A549 cells (**g**) at MOI of 30:1. Immunofluorescence staining was performed at 8 h post-infection using 4B11 monoclonal antibody specific to *B. pseudomallei* capsular polysaccharide to visualize the bacteria in green; phalloidin for F-actin in red; and Hoechst 33258 for the host DNA in blue. (**h**) The length of actin tails (white arrow) was determined using Zen Zeiss 3.0 SR (black) software tools. The mean lengths were: 3.1605 μm, BimA_Bp_ type 1; 1.988 μm, BimA_Bp_ type 2; 2.043 μm, BimA_Bp_ type 4; 1.807 μm, BimA_Bp_ type 6; 1.465 μm, BimA_Bp_ type 9; and 1.603 μm, BimA_Bp_ type 10 (one-way ANOVA; *P* < 0.05). Scale bar, 10 μm.

Since *B. pseudomallei* utilizes the BimA protein factor to polymerize host actin^17^, we performed immunostaining and confocal microscopy of A549 epithelial cells infected with the representative isolates of six BimA_Bp_ types to demonstrate their ability to form actin tails. The isolates included *B. pseudomallei* K96243 as representative of BimA_Bp_ type 1 and the reference strain; DR40130A, DR40111A, DR10008A, DR50173A, and DR50003A, representing BimA_Bp_ types 2, 4, 6, 9, and 10, respectively (Fig. 8a-g). The representative isolates of BimA_Bp_ types 2, 4, 6, and 9 carried the most distant BimA_Bp_ types from BimA_Bp_ type 1, and type 10 had a missense mutation in the transmembrane domain.

Our results demonstrated that all isolates were capable of polymerizing actin, as observed by the formation of a characteristic “comet” tail at the polar end of the bacteria. However, the mean and standard deviation (mean ± SD) actin tail length (µm) produced by the representative strains of BimA_Bp_ type 9 (1.465 ± 1.291, *P* = 0.0182) and type 10 (1.603 ± 1.211, *P* = 0.0391) were significantly shorter than those generated by the reference, BimA_Bp_ type 1 (*B. pseudomallei* K96243) (3.1605 ± 2.354). In contrast, the actin tail lengths (µm) produced by the representative strains of BimA_Bp_ type 2 (1.988 ± 1.627, *P* = 0.2227), type 4 (2.043 ± 1.701, *P* = 0.2714), and type 6 (1.807 ± 1.436, *P* = 0.1058) did not show significant differences compared to BimA_Bp_ type 1 (*B. pseudomallei* K96243) (Fig. 8h).

## Discussion

Previous studies have reported the presence of a BimA_Bm_ in *B. pseudomallei* isolates from Australia, India, and Sri Lanka and their association with neurological melioidosis^30–32^. However, the sequence variation within BimA_Bp_ and its impacts on the actin-based motility function of BimA are unexplored. Similarly, while the role of BimC in actin-based motility and *B. pseudomallei* pathogenesis has been reported^21^, its variants and their implications have not been well investigated.

In this study, we examined the genomes of 1,294 *B. pseudomallei* clinical isolates and observed that BimA and BimC exhibited variations. In addition to BimA_Bp_ type 1 of *B. pseudomallei* K96243, the BimA_Bp_ variants were further classified into nine types, and four types (BimA_Bp_ types 1,2, 3, and 4) were predominant in the three dominant lineages of *B. pseudomallei*, suggesting that BimA_Bp_ variations may make some contributions to the formation of dominant lineages. However, in vitro and in vivo experiments are warranted to validate this conclusion.

Our analysis of 1,294 genomes identified five major BimC types of which none possessed insertion or deletion sequences but harbored nonsynonymous SNPs, suggesting that BimC is more conserved than *bimA* in *B. pseudomallei*. Moreover, 45% of our isolates (585/1,294) fell under BimC type 1, which shares 100% of the sequences with BimC of the *B. pseudomallei* K96243. Furthermore, BimC type 1 was predominant in major lineage 1, while the remaining BimC variant types 2–5 were associated with the minor lineages. Although we could not find a BimA_Bm_ variant from our *B. pseudomallei* collections in Thailand, the dominant BimA_Bp_ and BimC variants were widely dispersed across northeast Thailand, which is an evolutionary and transmission hotspot for *B. pseudomallei* in Southeast Asia^52^.

3D structural modeling of BimA_Bp_ types allowed us to observe one or two additional PDAST (*p*roline, aspartic acid (*D*), *a*lanine, *s*erine, and *t*hreonine) repeat sequences, with observable changes of threonine to alanine in this region, from the usual five PDAST repeats of the *B. pseudomallei* K96243. Similarly, there were variations in the number of PDASX repeats in BimA, ranging from two (found in *B. pseudomallei* BCC215) to seven (*B. pseudomallei* MSHR305)^23^. Notably, these PDASX repeats are the predicted sites of host casein kinase 2 phosphorylation^23^, an important post-translational modification catalyzed by protein kinases to regulate cell processes in eukaryotes and bacteria^53^. Protein kinases usually phosphorylate bacterial proteins on serine and threonine amino acid residues to conform structural changes or modify protein–protein interactions^54^. An example of a protein kinase is YopO of *Yersinia* spp., whose phosphorylation of the host actin-modulating proteins disrupts the actin filaments and inhibits actin polymerization^55^. Furthermore, Sitthidet et al. have explored the impact of in-frame deletion of two, five, and seven PDASX repeats in BimA *B. pseudomallei*, wherein they found that increased PDASX repeats function additively, indicating an increased rate of actin polymerization and assembly^48^. In addition, the *trans*-complement of *B. pseudomallei bimA* mutant harboring BimA with two, five, and seven PDASX repeats could restore actin-based motility and plaque formation in A549 cells without discernible variations between the morphology of actin tails and plaques^48^. In-depth molecular and biochemical investigations on the implications of the phosphorylation of BimA will be needed to gain a deeper understanding of the actin dynamics and functions of BimA in *B. pseudomallei*.

Our study observed that the *B. pseudomallei* isolates, which harbored phylogenomically distant BimA_Bp_ types, formed plaques after 24 h of infection in A549 cells. However, the *B. pseudomallei* isolates carrying BimA_Bp_ type 9 formed significantly lower plaques. The variation in the results of plaque-forming efficiency assay might be due to other bacterial motility factors, like the *motA2* from the *fla2* flagellar cluster where the deletion of *bimA* and *motA2* genes almost completely abolished plaque formation in HEK293 cells^49^. To support our findings, we found that these strains do not only harbor variations in BimA_Bp_ but also in BPSL0097, BPSS0015, BPSS1494 (VirG), BPSS1495 (VirA), BPSS1498 (Hcp-1), BPSS1818 and BPSS1860, which have been described as being involved in actin-based functions in *B. pseudomallei*^18,49–51^. Moreover, we observed that the representative *B. pseudomallei* isolates with sequence variations within BimA_Bp_ were still effective in developing the actin tails. However, the mean actin tail length generated by BimA_Bp_ type 9 was significantly shorter, consistent with the observed lower plaque formation. These observations could be due to variations in regulatory gene *virAG*, which controls the expression of BimA^56^, or other proteins involved in the actin-development process. For instance, Jitprasutwit et al. identified a cellular protein, named ubiquitous scaffold protein Ras GTPase-activating-like protein (IQGP1) and showed that it was recruited to the infected actin tails of *B. pseudomallei* and controlled the length and density of the actin tails^57^. Furthermore, we found the variation in BPSS1818, a predicted inner membrane protein which modulates the host’s tubulin, suggesting that it might also influence the actin-tail formation and actin-based motility of *B. pseudomallei*^50^.

This study has some limitations. First, genomic analysis was only performed on our clinical isolates, mainly collected in northeast Thailand, which may not cover all isolates that may have the *bimA*_*Bm*_ gene in Southeast Asia. An In-depth screening for a *bimA*_*Bm*_ allele in Southeast Asia will benefit from a large-scale genomic analysis that covers all global *B. pseudomallei* clinical and environmental isolates. Second, the isolates used in our plaque, immunostaining, and confocal microscopy assays were merely representatives of the BimA_Bp_ variants, and each strain may employ various mechanisms and adaptive strategies to endow *B. pseudomallei* with virulence and overcome the host. More research into the BimA_Bp_ and BimC variants at the molecular and structural level is crucial to better understand the virulence, pathogenicity and implications of BimA_Bp_ and BimC variations.

Overall, this study highlights the variations within BimA_Bp_ and BimC and the implications of BimA_Bp_ variants for *B. pseudomallei* pathogenesis in A549 epithelial cells. Moreover, our work provides additional insight into the virulence mechanisms of *B. pseudomallei* and may aid in developing future research and therapeutic strategies for melioidosis.

## Materials and methods

### Biosafety approval

This study was approved by the Institutional Biosafety Committee of the Faculty of Tropical Medicine, Mahidol University (MU2022-028). All experiments were performed in accordance with relevant guidelines and regulations. All experiments involving *B. pseudomallei* were performed in a biosafety level 3 laboratory.

### Whole genome sequencing data

The data sets used in this study were 1,294 short-read genomes from our previous DORIM study^39^. The genomes utilized were from 1,294 *B. pseudomallei* clinical isolates (collected from melioidosis patients admitted to nine hospitals (Udon Thani Hospital,Khon Kaen Hospital, Srinakarind Hospital, Nakhon Phanom Hospital, Mukdahan Hospital, Roi Et Hospital, Surin Hospital, Sisaket Hospital, and Buriram Hospital) in northeast Thailand between July 2015 and December 2018)^38^. Of all genomes, 1,265, 27, and 2 were the *B. pseudomallei* genomes isolated from patients residing in northeast Thailand, Laos, and Cambodia, respectively. All genomes were checked for potential contamination with other closely related species by assigning taxonomic identity using Kraken v.1.1.1^58^. Epidemiological data, isolate data, and genome accession codes used in this study are listed in Supplementary Data 1.

### Genome assembly and mapping alignment

We performed *de novo* assembly of short-read data using Velvet v.1.2.10 ^59^. Genome alignment of the 1,294 isolates used in this study and an Australasian outgroup MSHR5619 was achieved by mapping short-read sequences to *B. pseudomallei* strain K96243 (accession numbers BX571965 and BX571966) using Snippy v.4.6.0 (https://github.com/tseemann/snippy). To prevent mapping errors and false SNP identifications, we filtered out SNPs with coverage of fewer than 10 reads and frequency below 0.9.

### Detection of variations in BimA and BimC

*bimA*_*Bp*_ (BPSS1492 in *B. pseudomallei* K96243), *bimA*_*Bm*_ (BURPS668_A2118 in *B. pseudomallei* MSHR668), and *bimC* (BPSS1491 in *B. pseudomallei* K96243), were determined in 1,294 assembled genomes using BLAST with the option to retrieve *bimA*_*Bp*,_*bimA*_*Bm*_, and *bimC* sequences from each genome. Nucleotide alignment was subjected to CD-HIT v.4.8.1 ^60^ with a 100% threshold to identify variations. Nucleotide sequences of each *bimA* and *bimC* types were then translated into amino acid sequences using the Sequence Manipulation Suite translation tool^61^ (www.bioinformatics.org) and aligned using MAFFT v.7 ^62^. All *bimA* variants were subjected to PCR and were confirmed by DNA sequencing. Pairwise SAP (single amino acid polymorphism) distances were calculated using snp-dists v.0.7.0 (https://github.com/tseemann/snp-dists). SAP is defined based on single substitution of amino acid from the reference strain *B. pseudomallei* K96243.

Additionally, detection of variations in *bpsl0097*,* bpss0015*, *virG* (BPSS1494 in *B. pseudomallei* K96243), *virA* (BPSS1495 in *B. pseudomallei* K96243), *hcp-1* (BPSS1498 in *B. pseudomallei* K96243), *bpss1818*, and *bpss1860* in the genomes used in plaque-forming efficiency assay (DR10025A, DR20021A, DR40130A, DR40111A, DR80025A, DR90085A, DR10008A, DR40025A, DR50053A, DR50173A, DR70003A, DR90006A, DR20062A, DR50003A, and DR60054A) were performed following the same method as the detection of variations in BimA and BimC.

### Population structure analysis

Of the 1,294 *B. pseudomallei* genomes used in our study, 1,265 isolates were studied for the population structure by our previous project^39^. In this study, we re-analyzed the population structure by adding 29 *B. pseudomallei* genomes from Laos (*n* = 27) and Cambodian (*n* = 2) patients to the 1,265 genomes using a combination of two independent approaches: (i) employing PopPUNK v.2.4.0 ^63^ and (ii) constructing the ML phylogeny of core-SNP alignment with IQ-TREE v.2.0.3 ^64^ as methods described by Seng et al.^39^. The spatial distribution map of the dominant lineages and the BimA_Bp_ and BimC types were plotted using the latitude and longitude of the patients’ home addresses.

### 3D protein structure modeling

BimA and BimC amino acid sequences were subjected to BLAST search against the Protein Data Bank (PDB) (http://www.rcsb.org) to find a template for homology modeling. For BimA, our search in SWISS-MODEL yielded models with low percent sequence identity and low coverage (< 40%)^46^, which were unsuitable. We then proceeded to Phyre 2, an automatic fold recognition server for predicting the structure and/or function of target protein sequence, to enhance the search^41^, but we obtained similar results. We also explored AlphaFold; however, although the model generated had high sequence coverage (> 90%), our residues of interest (175–263) were in a region with a very low confidence score (pLDDT < 50)^65^. Therefore, we used the *de novo* protein modeling tool, I-TASSER (Iterative Threading ASSEmbly Refinement)^42^, which works through threading, to generate the best model for BimA. I-TASSER produced model 1 with a confidence score (C-score) of -0.96. C-score is calculated based on the significance of threading template alignments and the convergence parameters of the structure assembly simulations. C-score typically ranges from − 5 to 2, with higher value indicating models of greater confidence and vice-versa^42^. The BimA model built by I-TASSER was then subjected to the YASARA energy minimization tool^43^ and validated using the SAVES PROCHECK server (https://saves.mbi.ucla.edu/). All ten BimA_Bp_ type models were built on one reference model BimA_Bp_ K96243. The BimA_Bm_ model was built using the reference BimA_Bm_ MSHR668 with a confidence score of -0.93.

The BimC protein model was built based on a reference in PDB using SWISS-MODEL^46^, using the template TibC, a dodecameric iron-containing heptosyltransferase from enterotoxigenic *E. coli* H10407 (4RB4), which has 43.85% sequence identity and 93% coverage of the BimC of *B. pseudomallei* K96243. The built model has a GMQE score of 0.77 ^46^ and validated using the SAVES PROCHECK server (https://saves.mbi.ucla.edu/). The generated 3D structures of the models were visualized using Discovery Studio Visualizer software (Biovia v.21.1).

### Plaque-forming efficiency assay

The plaque-forming efficiency was evaluated using A549 epithelial lung cells (CCL-185, American Type Culture Collection, MD, USA) as described previously^66^ with some modifications for 16 representative strains of the BimA_Bp_ type 1 (*B. pseudomallei* K96243), type 2 (DR10025A, DR20021A and DR40130A), type 4 (DR40111A, DR80025A and DR90085A), type 6 (DR10008A, DR40025A and DR50053A), type 9 (DR50173A, DR70003A and DR90006A) and type 10 (DR20062A, DR50003A and DR60054A). The cells were seeded at 3.0 × 10^5^ cells/well into a 24-well tissue culture plate and incubated at 37℃ with 5% CO_2_ overnight. The culture medium was replaced with fresh RPMI 1640 medium (Gibco BRL, Grand Island, NY, USA) supplemented with 10% heat-inactivated fetal bovine serum (FBS) (HyClone, USA) to prepare the cells for infection. The cells were infected with representative strains of the BimA_Bp_ variant types in duplicates at a multiplicity of infection (MOI) of 0.1:1 at 37℃ with 5% CO_2_ for 2 h. Thereafter, the infected cell monolayers were washed with PBS two times and maintained in a culture medium containing 250 µg/ml kanamycin (Invitrogen) for 24 h to eliminate the extracellular bacteria. The infected cells were fixed with 4% formaldehyde and stained with 2% (w/v) crystal violet for 2 min. The plaque confirmatory test was done by visualizing the plaques under the microscope. Plaque-forming efficiency (PFU/ml) was counted as the number of plaques (plaque-forming units: PFU) formed, divided by the inoculation volume of bacteria (CFU/ml). The plaque-forming efficiency assay was performed in three independent experiments.

### Immunostaining and confocal microscopy

Immunostaining was performed for six representative isolates of BimA_Bp_ type 1 (*B. pseudomallei* K96243, classified as type 1 was used as a reference strain), type 2 (DR40130A), type 4 (DR40111A), type 6 (DR10008A), type 9 (DR50173A), and type 10 (DR50003A) as previously described^66^ with some modifications. Briefly, A549 cells were seeded at 1.5 × 10^5^ cells/well on a sterile glass coverslip in a six-well tissue culture plate and incubated overnight at 37℃ with 5% CO_2_. The monolayers were infected with the six representative isolates at an MOI of 30:1 for 2 h. Subsequently, the cells were washed with PBS three times, and the extracellular bacteria were killed with 250 µg/ml kanamycin in RPMI. The infected cells were incubated further for 6 h, washed with PBS three times, fixed with 4% paraformaldehyde in PBS for 30 min, and permeabilized with 0.5% triton X-100 for 30 min. After the washing, the permeabilized cells were incubated with 1:200 of 4B11 (2.5 µg/ml) monoclonal antibody specific to *B. pseudomallei* capsular polysaccharide^67^ at 37℃ for 1 h. The cells were then washed with PBS three times, followed by incubation with goat anti-mouse IgG conjugated with Alexa Fluor 488 at dilution of 1:1,000 (Invitrogen) for *B. pseudomallei* detection, Alexa Fluor 647-conjugated phalloidin at dilution of 1:1,000 (Invitrogen) for actin staining, and Hoechst 33258 (1:1,000) (Invitrogen) for nuclear staining at 37℃ for 1 h. The stained cells were washed with PBS three times, and the coverslips were mounted on glass slides using 8 µl of ProLong Gold antifade reagent (Invitrogen). Confocal microscopy was performed with a laser scanning confocal microscope (LSM 700; Carl Zeiss) using 100× objective lenses with oil immersion and Zen software (2010 edition, Zeiss, Germany). The excitation and emission wavelengths were 496/519 for Alexa Fluor 488, 352/461 for Hoechst 33258, and 594/633 for Alexa Fluor 647. For actin tail length measurement, Zeiss Zen 3.0 SR (black) software tools were utilized by counting 20 bacteria with actin tail per representative image of the BimA_Bp_ type-harboring strains.

### Statistical analysis

Statistical analysis was performed using GraphPad Prism software version 9.0 (GraphPad Software Inc, La Jolla, CA). The data are presented as individual points and mean ± SD. One-way ANOVA was used to compare three or more groups, while the *t*-test was used to compare two groups. For comparisons involving the lineage distribution of BimA_Bp_ and BimC types, Chi-square test was used. The results were considered statistically significant at *P* < 0.05.

### Ethical approval

This study and the consent procedure were approved by the Ethics Committee of the Faculty of Tropical Medicine, Mahidol University (MUTM 2015-002-01 and MUTM 2022-038-01). All research was performed in accordance with relevant guidelines and regulations. Written informed consent was obtained from all participants or their representatives before enrollment.

## Electronic supplementary material

Below is the link to the electronic supplementary material.

Supplementary Material 1

Supplementary Material 2

Supplementary Material 3

Supplementary Material 4

Supplementary Material 5

Supplementary Material 6

## Data Availability

The genome sequence data presented in this study can be found in online repositories. The ENA under study accession number PRJEB25606 and PRJEB35787. The accession numbers for individual genomes were listed in Supplementary Data 1.
