# Supplementary material for: Genetic variation, structural analysis, and virulence implications of BimA and BimC in clinical isolates of Burkholderia pseudomallei in Thailand
Source: Sci Rep. 2024 Oct 23;14:24966. doi: 10.1038/s41598-024-74922-3 (PMC11499645; doi:10.1038/s41598-024-74922-3)
Supplement: Supplementary file 1 — Supplementary Material 1 [file 41598_2024_74922_MOESM1_ESM.pdf]

Genetic variation, structural analysis, and virulence implications of BimA and BimC in clinical isolates of *Burkholderia pseudomallei* in Thailand

Charlene Mae Salao Cagape, Rathanin Seng, Natnaree Saiprom, Sarunporn Tandhavanant, Claire Chewapreecha, Usa Boonyuen, T. Eoin West, and Narisara Chantratita

Corresponding Author: Narisara Chantratita, e-mail: narisara@tropmedres.ac, Tel: (+66) 819099772

This document provides information on Supplementary Data and Supplementary Table.

Supplementary Data 1 Epidemiological data, accession codes, $BimA_{Bp}$ and BimC types of genomes used in this study. The accession codes for raw sequence reads used in this study were deposited in the European Nucleotide Archive (ENA) (n = 1,294). The PopPUNK lineage data were previously described by Seng et al., 2023. (Provided as a separate excel file).

Supplementary Data 2 Variations in *B. pseudomallei* isolates used in plaque assay (provided as a separate excel file).

Supplementary Data 3 Amino acid sequences of BimA types (provided as a separate txt file).

Supplementary Data 4 Amino acid sequences of major BimC types (provided as a separate txt file).

Supplementary Table S1 Lineage distribution of $BimA_{Bp}$ and BimC types in the 1,294 *B. pseudomallei* clinical isolates (provided as a separate word file).
