# Supplementary material for: Genetic variation, structural analysis, and virulence implications of BimA and BimC in clinical isolates of Burkholderia pseudomallei in Thailand
Source: Sci Rep. 2024 Oct 23;14:24966. doi: 10.1038/s41598-024-74922-3 (PMC11499645; doi:10.1038/s41598-024-74922-3)
Supplement: Supplementary file 6 — Supplementary Material 6 [file 41598_2024_74922_MOESM6_ESM.docx]

|  |  | | |  |  | |  |
| --- | --- | --- | --- | --- | --- | --- | --- |
|  | **No. (%) of Bp genomes** | | |  | **Chi-square test**  **(Lineages 1, 2 and 3)** | | |
|  |  | | |  |  | |  |
| **Types of BimA and BimC** | **Lineage 1 (n = 317)** | **Lineage 2 (n = 271)** | **Lineage 3 (n = 113)** | **Non-dominant lineages 4 – 101 (n = 593)** |  | **Value, df** | **P value** |
| **BimA_Bp_** |  |  |  |  |  |  |  |
| **1** | 174 (55) | 24 (9) | 37 (33) | 295 (50) |  | 139.0, 2 | <0.0001 |
| **2** | 0 (0) | 224 (83) | 0 (0) | 7 (1) |  | 522.3, 2 | <0.0001 |
| **3** | 8 (3) | 2 (1) | 68 (60) | 86 (15) |  | 328.2, 2 | <0.0001 |
| **4** | 118 (37) | 6 (2) | 2 (2) | 7 (1) |  | 145.5, 2 | <0.0001 |
| **5** | 0 (0) | 1 (0) | 1 (1) | 47 (8) |  | — | — |
| **6** | 0 (0) | 0 (0) | 0 (0) | 37 (6) |  | — | — |
| **7** | 1 (0) | 0 (0) | 0 (0) | 34 (6) |  | — | — |
| **8** | 0 (0) | 0 (0) | 0 (0) | 7 (1) |  | — | — |
| **9** | 0 (0) | 0 (0) | 0 (0) | 5 (1) |  | — | — |
| **10** | 0 (0) | 0 (0) | 0 (0) | 4 (1) |  | — | — |
| **N/A** | 16 (5) | 14 (5) | 5 (4) | 64 (1) |  | — | — |
| **BimC** |  |  |  |  |  |  |  |
| **1** | 284 (90) | 75 (28) | 53 (47) | 173 (29) |  | 239.0, 2 | <0.0001 |
| **2** | 20 (6) | 92 (34) | 54 (48) | 287 (48) |  | 105.1, 2 | <0.0001 |
| **3** | 8 (3) | 88 (32) | 6 (5) | 97 (16) |  | 114.6, 2 | <0.0001 |
| **4** | 3 (1) | 3 (1) | 0 (0) | 15 (3) |  | — | — |
| **5** | 1 (0) | 0 (0) | 0 (0) | 3 (1) |  | — | — |
| **Minor types** | 1 (0) | 13 (5) | 0 (0) | 18 (3) |  | 17.74, 2 | 0.0001 |

**Supplementary Table S1. Lineage distribution of BimA_Bp_ and BimC types in the 1,294 *B. pseudomallei* clinical isolates**

Chi-square test was performed using GraphPad Prism software version 9.0 (GraphPad Software Inc, La Jolla, CA) to compare the lineage distribution of BimA_Bp_ and BimC types in the 1,294 *B. pseudomallei* clinical isolates. N/A indicates the genomes in which *bimA_Bp_* genes are fragmented. Minor types are the 25 minor BimC types (types 6–30). — indicates Chi-square test was not performed. df, degrees of freedom. The results were considered statistically significant at P <0.05.
